# Involvement of the Cytokine MIF in the Snail Host Immune Response to the Parasite *Schistosoma mansoni*

**DOI:** 10.1371/journal.ppat.1001115

**Published:** 2010-09-23

**Authors:** Alvaro Baeza Garcia, Raymond J. Pierce, Benjamin Gourbal, Elisabeth Werkmeister, Dominique Colinet, Jean-Marc Reichhart, Colette Dissous, Christine Coustau

**Affiliations:** 1 Institut Pasteur de Lille, CIIL, Lille, France; Inserm, U 1019, Lille, France; Univ Lille Nord de France, Lille, France; CNRS, UMR 8204, Lille, France; 2 UMR 5244 CNRS-EPHE- UPDV, Université Perpignan Via Domicia, Perpignan, France; 3 MICPaL Facility IFR 142-CNRS UMR 8161, Institut Pasteur de Lille, Lille, France; 4 UMR 6243 INRA-CNRS-UNSA, Centre Sophia Agrobiotech, Sophia Antipolis, France; 5 UPR 9022 CNRS, Institut de Biologie Moléculaire et Cellulaire, Strasbourg, France; NIAID/NIH, United States of America

## Abstract

We have identified and characterized a Macrophage Migration Inhibitory Factor (MIF) family member in the Lophotrochozoan invertebrate, *Biomphalaria glabrata*, the snail intermediate host of the human blood fluke *Schistosoma mansoni*. In mammals, MIF is a widely expressed pleiotropic cytokine with potent pro-inflammatory properties that controls cell functions such as gene expression, proliferation or apoptosis. Here we show that the MIF protein from *B. glabrata* (BgMIF) is expressed in circulating immune defense cells (hemocytes) of the snail as well as in the *B. glabrata* embryonic (Bge) cell line that has hemocyte-like features. Recombinant BgMIF (rBgMIF) induced cell proliferation and inhibited NO-dependent p53-mediated apoptosis in Bge cells. Moreover, knock-down of BgMIF expression in Bge cells interfered with the *in vitro* encapsulation of *S. mansoni* sporocysts. Furthermore, the *in vivo* knock-down of BgMIF prevented the changes in circulating hemocyte populations that occur in response to an infection by *S. mansoni* miracidia and led to a significant increase in the parasite burden of the snails. These results provide the first functional evidence that a MIF ortholog is involved in an invertebrate immune response towards a parasitic infection and highlight the importance of cytokines in invertebrate-parasite interactions.

## Author Summary

*Schistosoma mansoni*, a parasitic blood fluke that causes intestinal schistosomiasis in humans, requires an intermediate host, a freshwater snail of the genus *Biomphalaria* for its transmission. Infection of the snail triggers marked cellular and humoral immune responses, but the molecular mechanisms of these responses are not well known. We have identified and characterized the involvement of a snail homologue of the cytokine MIF (Macrophage Migration Inhibitory Factor) in the snail immune responses to infection by the parasite. By using biochemical and molecular approaches in combination with *in vitro* culture and *in vivo* gene knock down, we have demonstrated the role of snail MIF in the regulation of the snail innate immune system. In particular, MIF regulates the proliferation and activation of the hemocytes, the macrophage-like snail defense cells, and the encapsulation response. This shows for the first time that MIF has a conserved cytokine function in an invertebrate and underlines the interest of the schistosome-snail model in the study of innate immunity.

## Introduction

Schistosomiasis, the second most widespread human parasitic disease after malaria [1], is caused by helminth parasites of the genus *Schistosoma* and more than 200 million people in 74 countries suffer from the pathological consequences of this disease [2]. Human infection requires contact with freshwater in which infected snails (the intermediate hosts of schistosomes) have released cercariae larvae that penetrate human skin. The complex interaction between the intermediate snail host and the parasite and in particular between *Schistosoma mansoni* and the snail generally used for its laboratory maintenance, *Biomphalaria glabrata*, is of interest both in terms of transmission dynamics, but increasingly as a model for the study of the innate immune response and its evolution.

In order to protect themselves against pathogens, invertebrates use innate immune responses such as wound repair, coagulation, phagocytosis and encapsulation reactions, also used by vertebrates [3]. Major signalling pathways or effector molecules underlying innate immune responses of vertebrates and invertebrates are also shared, as for instance the Toll receptors described for the first time in *Drosophila* [4] or members of immunoglobulin superfamily such as the FREPs (Fibrinogen-RElated Proteins) in *B. glabrata* [5].

The need for regulation of cellular immunity and the parallels made between vertebrate and invertebrate innate immunity led to an intense search for invertebrate cytokines [6]. Cytokines specific to invertebrates, such as spätzle in *Drosophila* [4], astakine in *Pacifastacus leniusculus* [7], or CCF in *Eisenia foetida* [8] have been described, but to date, only very few orthologs of vertebrate cytokines have been incontrovertibly identified in invertebrates [9], one of which is Macrophage Migration Inhibitory Factor (MIF). MIF was one of the first mammalian cytokines to be discovered and has been described as a pivotal regulator of innate immune and inflammatory responses in mammals [10]. It was first characterized as a factor derived from activated T cells that inhibited random migration of macrophages [11], [12]. Since the first cloning of a MIF gene [13] many biological activities have been described, including stimulation of cell proliferation through ERK1/ERK2 pathway activation, activation of the response against endotoxin or Gram negative bacteria by upregulation of TLR4 (the signal-transducing molecule of the LPS receptor complex) expression and the suppression of p53-mediated growth arrest in macrophages challenged by LPS [10]. In addition MIF possesses intrinsic tautomerase activity (keto-enol isomerisation of small aromatic substrates such as L-dopachrome methyl ester) that is dependent on the post-translational cleavage of the initiating methionine to expose an N-terminal proline residue [14].

Interestingly, MIFs have been characterized in a wide variety of parasites, including nematodes and protozoans [15], [16] but the role of the cytokine has been mainly studied in the context of the host-parasite relationship with the emphasis on the effect of parasite MIF on the host immune response. For instance *Ancylostoma* MIF has been shown to bind to the human MIF receptor [17] and recombinant *Brugia* MIF induces the release of cytokines (IL-8, TNFα) from human macrophages [18]. Similarly, *Plasmodium* MIF is thought to influence the host immune response and the course of anemia during infection [16]. MIFs have also recently been identified in two species of mollusks, disk abalones [19], but currently, nothing is known about the role of MIF from the invertebrate host during an immune response to a pathogen. Strikingly, an exhaustive search of the *S. mansoni* genomic sequences (AB-G, unpublished) failed to find any MIF signature sequences These *in silico* findings are consistent with the *in vitro* work of others describing the absence of MIF homologs in parasitic trematodes [20].

The discovery in *B. glabrata* of a potential cytokine-like molecule displaying significant sequence similarity to MIF [21], raised the question of its potential involvement in the regulation of the snail immune response to parasite infection. In this report, we demonstrate that the MIF protein from *B. glabrata* (BgMIF) is expressed in circulating immune defense cells (hemocytes) of the snail as well as in the *B. glabrata* embryonic (Bge) cell line that has hemocyte-like features. We show that recombinant BgMIF (rBgMIF) possesses the conserved tautomerase enzymatic activity of the MIF family, induces cell proliferation (correlating with ERK phosphorylation) and inhibits NO-dependent, p53-mediated apoptosis in Bge cells. Moreover, knock-down of BgMIF in Bge cells inhibits the *in vitro* encapsulation of *S. mansoni* sporocysts and this correlates with an inhibition of p38 phosphorylation in these cells. Finally, in whole snails, we demonstrate the involvement of BgMIF in the snail anti-parasitic response towards *S. mansoni*. Furthermore, the tools developed here pave the way toward a better understanding of the complex interactions between *S. mansoni* and its molluscan snail host.

## Results

### The freshwater snail *B. glabrata* expresses a MIF ortholog with tautomerase activity

**Figure 1:**
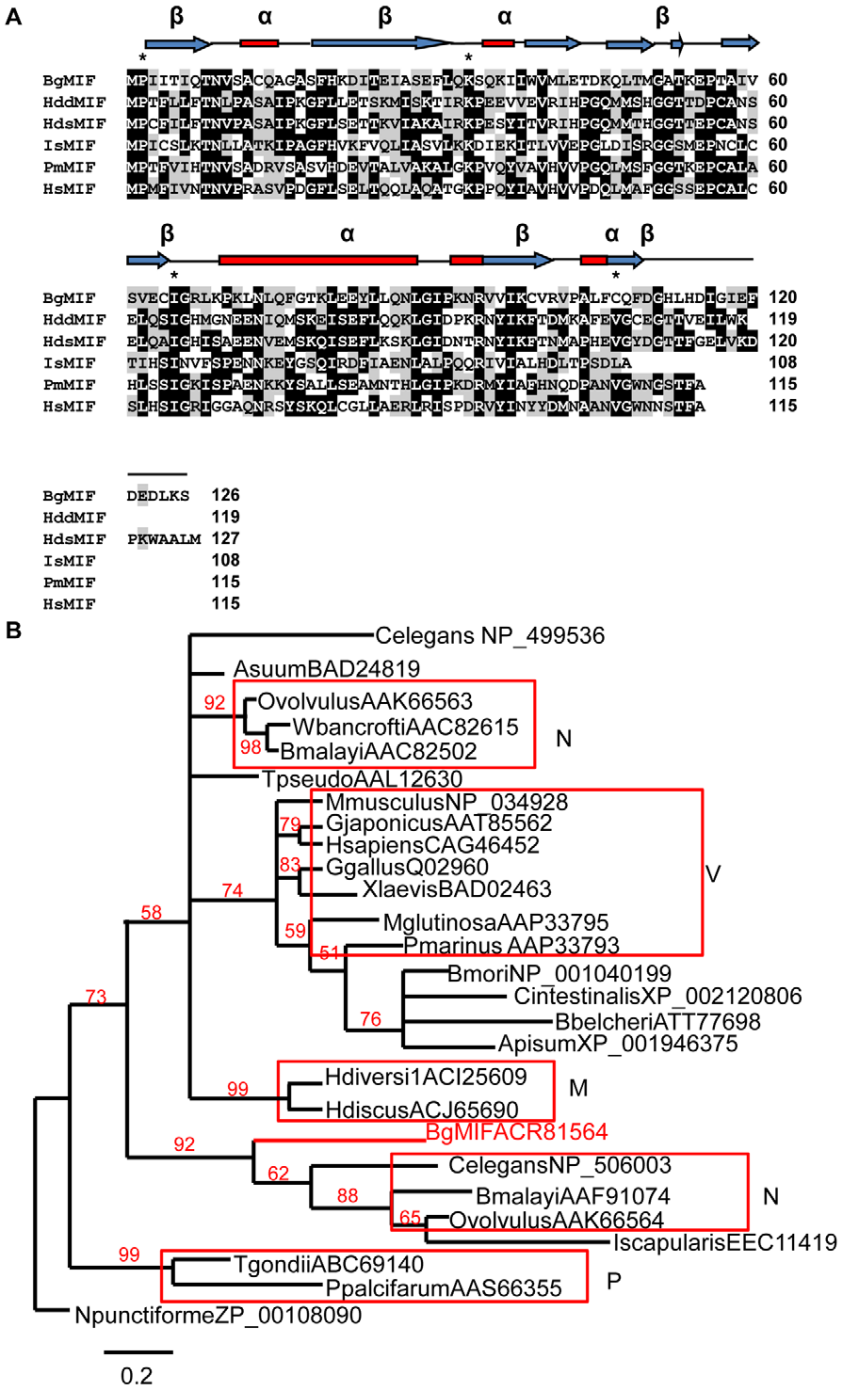
BgMIF is a variant member of the MIF family. (A) Alignment of the BgMIF (BgMIF; accession number: ACR81564) peptide sequence to MIFs from *Haliotis discus discus* (HddMIF; accession number: FJ435176), *Haliotis diversicolor sextus* (HdsMIF; EU284114), *Ixodes scapularis* (IsMIF; EEC11419), *Petromyzon marinus* (PmMIF; accession number: AA833793) and *Homo sapiens* (HsMIF; accession number: NM_002415). Residues conserved in the tautomerase catalytic site are marked with a star. Putative protein secondary structure (α = α-helix, β = β-sheet) according to (11) is shown above the sequence alignment. (B) Maximum-likelihood tree of BgMIF and MIF proteins from selected species (Genbank accession numbers and name of species are shown on the tree). The tree obtained using MrBayes is shown (see methods). Numbers above branches are Bayesian Posterior Probability values (100000 replicates). Horizontal distances are proportional to percents of divergence between tip species and nodes (scale given), vertical distances are arbitrary. Only BPP values >0.5 are shown. M, Mollusks; N, Nematodes; P, Protozoans; V, Vertebrates.

Alignment of MIF peptide sequences (Figure 1A) shows that BgMIF contains the N-terminal catalytic proline (Pro2) that is exposed by cleavage of the initiating methionine and is essential for tautomerase activity (see below and [14]). With 31% sequence identity to human MIF, BgMIF is less conserved than MIFs from two other mollusks, the bivalve abalones, *Haliotis diversicolor sextus* (39%) and *Haliotis discus discus* (35%). Several invariant active site residues [15] are conserved, including Lys32 and Ile64. The conserved Val106 residue is substituted by a Cys in BgMIF or by Leu in MIF from *Ixodes scapularis,* thus maintaining the presence of a hydrophobic residue at this position (Figure 1A). To further investigate the relationship between BgMIF and other MIFs, we performed a phylogenetic analysis (using two different analyses with similar results: see Methods) on selected vertebrate and invertebrate proteins (Figure 1B). The phylogeny of selected MIFs proved to be complex with numerous small clades and no strong relationship with taxonomy. Although BgMIF is clearly grouped in the phylogenetic tree with nematode MIF2 sequences [15], it is not closely related to other mollusk MIFs (Figure 1B).

**Figure 2:**
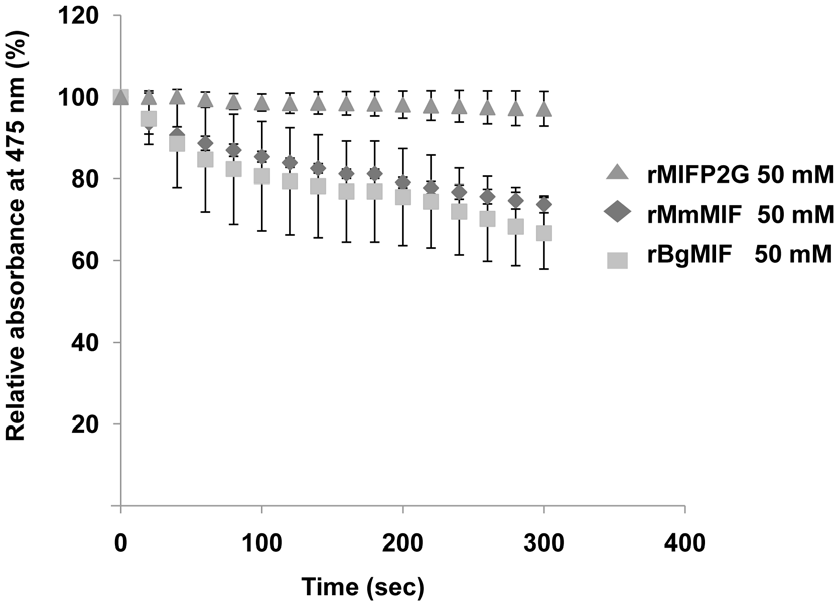
D-dopachrome tautomerase activity. Analysis of the D-dopachrome tautomerase enzymatic activity measured by loss in absorbance at 475 nm and plotted against the concentration of 2-carboxymethylester-2,3-dihydroindole-5,6-quinone for 50 nM wild type rBgMIF and mutated rBgMIF P2G. MmMIF at the same concentration was used as a positive control. Results shown are the means +/− S.D. of three independent experiments.

A hallmark of all MIF family members is the enzymatic tautomerase activity; we expressed it as a recombinant protein (rBgMIF) in *E. coli* together with a site-directed mutant (rBgMIFP2G), in which the N-terminal Proline (Pro2) was substituted by Gly. We used rBgMIF and rBgMIFP2G to perform a tautomerase assay with mouse MIF (rMmMIF) as a positive control and L-dopachrome methyl ester as a substrate. The results (Figure 2) showed that rBgMIF displayed tautomerase activity comparable to that of the mouse MIF protein and that, as expected; the mutant rBgMIFP2G did not have any detectable activity. Therefore, as in all MIF family members, Pro2 is required for enzymatic activity of BgMIF. In addition we tested the inhibition of the tautomerase activity using the MIF antagonist ISO-1 a specific inhibitor of mammalian MIF [22]. rBgMIF treated with 100 or 200 µM of ISO-1 (Supplementary data Fig S1) was inhibited by more than 95% at both doses.

### BgMIF is expressed by snail immune defense cells and secreted in the hemolymph

**Figure 3:**
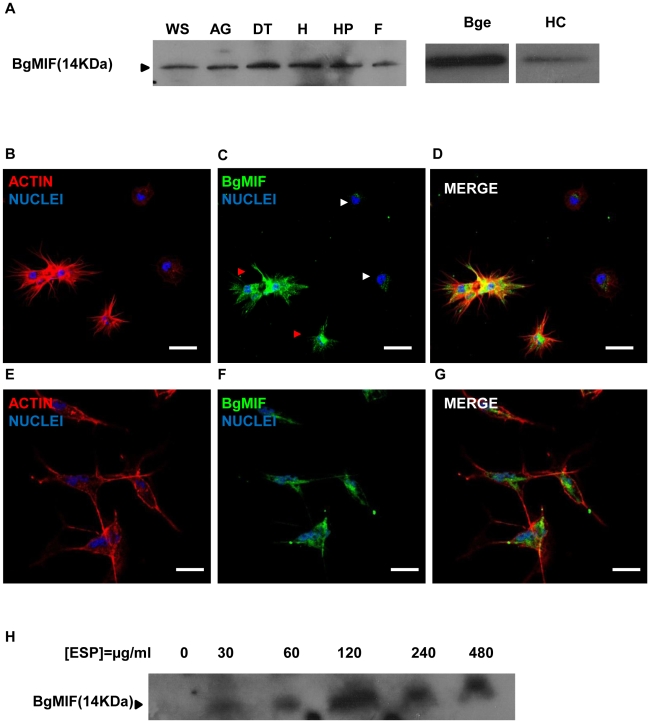
Expression of BgMIF. (A) BgMIF is detectable in various snail tissues. Western blotting using an antiserum directed against a peptide derived from BgMIF detects the protein as a single band in whole snails (WS), albumen gland (AG), digestive tract (DT), heart (H), hepatopancreas (HP), and foot (F), as well as in hemocytes (HC) and Bge cells (Bge). (B–D) Immunolocalization of BgMIF in hemocytes. Hemocytes were labeled with phalloidin (actin label) and Hoechst (B), with anti-BgMIF and Hoechst (C) or all three labels (D). Note that BgMIF labeling is much less visible in two unspread hemocytes (white arrows) compared to spread hemocytes exhibiting pseudopods (red arrows). Scale bar represents 12 µm. (E–G) Immunolocalization of BgMIF in Bge cells. Cells were labeled with phalloidin (actin label) and Hoechst (E), with anti-BgMIF and Hoechst (F) or all three labels (G). Scale bar represents 12 µm. (H) Immunoblotting of Bge cell culture supernatants with anti-BgMIF antibody. Cells were exposed to increasing concentrations of *S. mansoni* ESP (30–480 µg/mL).

In contrast to most cytokines, MIF is constitutively expressed and stored in intracellular pools. MIF secretion is induced by inflammatory stimuli such as endotoxin (LPS) or tumor necrosis factor (TNF-α), as well as by hormones [10]. MIF is expressed by defense cells such as macrophages [23], monocytes, neutrophils, dendritic cells and other cell types in tissues in contact with the host's natural environment [10]. We examined tissue specific expression of MIF in *B. glabrata* snails by western blotting of protein extracts from various snail organs using an antiserum raised against two peptides derived from the BgMIF sequence. This antiserum was shown to recognize native BgMIF (Figure 3A). A single band corresponding to BgMIF was found in all tissues tested, including the albumen gland, digestive tract, heart (hematopoietic organ) hepatopancreas, and foot (Figure 3A). In order to confirm the presence of BgMIF in *B. glabrata* hemocytes, we performed both western blotting and immunolocalisation analyses. BgMIF was detected in hemocyte lysates (Figure 3A) and immunolocalized in the cytoplasm of hemocytes. BgMIF was found to be more abundant in well spread hemocytes, termed granulocytes, than in unspread hemocytes or hyalinocytes [24], [25] (Figure 3B–D).

ELISA tests performed with anti-BgMIF serum allowed us to detect BgMIF in plasma (cell-free hemolymph) and to demonstrate that the amount of BgMIF in plasma progressively decreased during infection by *S. mansoni* (34% of decrease at 48 h post-infection) (Supplementary data Figure S2).

### BgMIF is expressed and secreted in the Bge cell line

Bge cells represent the only existing molluscan cell line and display hemocyte-like immune functions [26]. They have previously been described to share with hemocytes a fibroblastic origin and the ability to recognize and phagocyte or encapsulate foreign material including larval trematodes [26], [27]. In order to assess the pertinence of this cell line as an *in vitro* system for the analysis of BgMIF activity and function, we first searched for the presence of BgMIF in Bge cells. BgMIF was readily detectable in these cells by western blotting (Figure 3A) and immunolocalization showed that, as in hemocytes, BgMIF could be detected in the Bge cell cytoplasm (Figure 3E–G). In order to determine whether Bge cells could also release BgMIF protein upon immune stimulation, as observed *in vitro* for mammalian macrophages [23], we cultured Bge cells in the presence of *S. mansoni* excretory-secretory products (ESP) that have been shown to modulate gene expression in these cells [28]. BgMIF secretion was induced by ESP at 30 µg/mL (protein) with an apparent maximum at a dose of 120 µg/mL (Figure 3H).

We have also carried out a western blot of ESP from sporocysts with the anti-BgMIF antiserum and as expected in view of the absence of MIF signature sequences from the *S. mansoni* genome, no cross-reactive bands were detected (data not shown).

### rBgMIF stimulates cell proliferation by sustained activation of ERK in Bge cells

**Figure 4:**
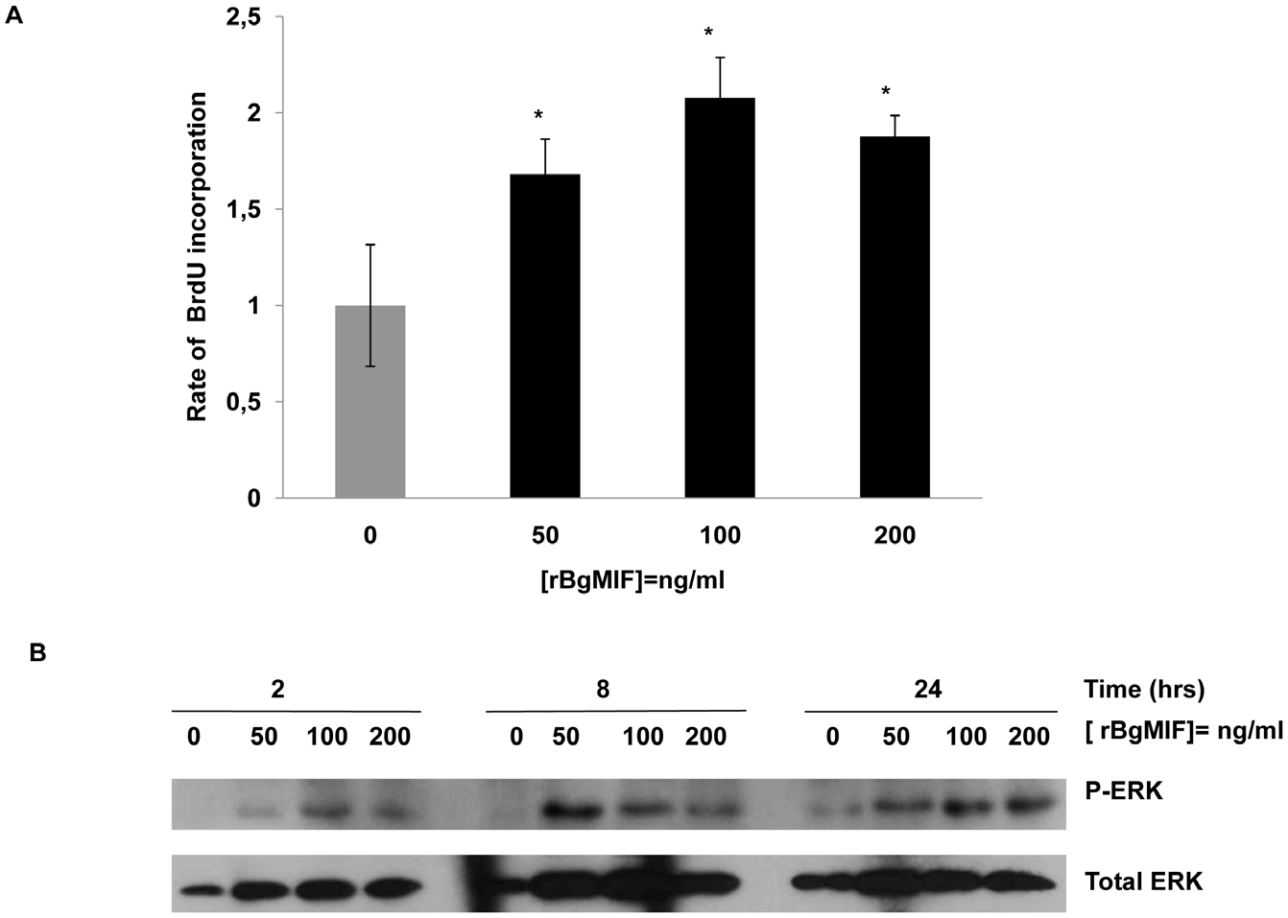
rBgMIF stimulates the proliferation and sustained activation of ERK in Bge cells. (A) Bge cell proliferation rate after exposure to various concentrations of rBgMIF. Proliferation was assessed using BrdU incorporation measured by an ELISA assay. Results are represented as fold increase in BrdU incorporation as compared to incorporation in control cells. The results shown are the mean ± SD of two assays carried out in quadruplicate and are representative of 3 separate experiments *p<0,05. (B) Immunoblotting of cell lysates using an anti-phosphorylated ERK 1/2 antibody (P-ERK) and an anti-ERK 1/2 antibody for control of total ERK content (total ERK).

In mammals, MIF stimulates the proliferation of quiescent fibroblasts in an “ERK sustained activation” dependent manner [29]. We determined whether BgMIF promotes cell proliferation by stimulating quiescent Bge cells with rBgMIF for 24 h and measuring BrdU incorporation. Purified rBgMIF stimulated cell proliferation in a dose dependent manner from a concentration of 50 ng/ml, with a maximum incorporation rate at 100 ng/ml (Figure 4A).

ERK-MAPK pathway activation is associated with mammalian MIF induced cell proliferation. To investigate whether BgMIF activated ERK, quiescent Bge cells were treated with rBgMIF and the cell lysates examined for ERK phosphorylation by Western blot analysis using phospho-specific anti-ERK antibodies. MIF induced phosphorylation of a *B. glabrata* ERK homolog in a dose and time-dependent fashion (Figure 4B). ERK phosphorylation was detected as early as 2 h and was sustained for at least 24 h as previously described for ERK in the NIH/3T3 fibroblast cell line (Figure 4B) [29]. In addition, U0126 the specific inhibitor of MEK (mitogen-activated protein kinase/ERK kinase), the upstream kinase of ERK [30], prevented the stimulatory effect of BgMIF on Bge cell proliferation (Supplementary data Figure S3) and ERK phosphorylation (data not shown), further indicating that MIF can induce proliferation via the ERK1/ERK2 pathway.

### rBgMIF suppresses NO induced apoptosis in Bge cells

**Figure 5:**
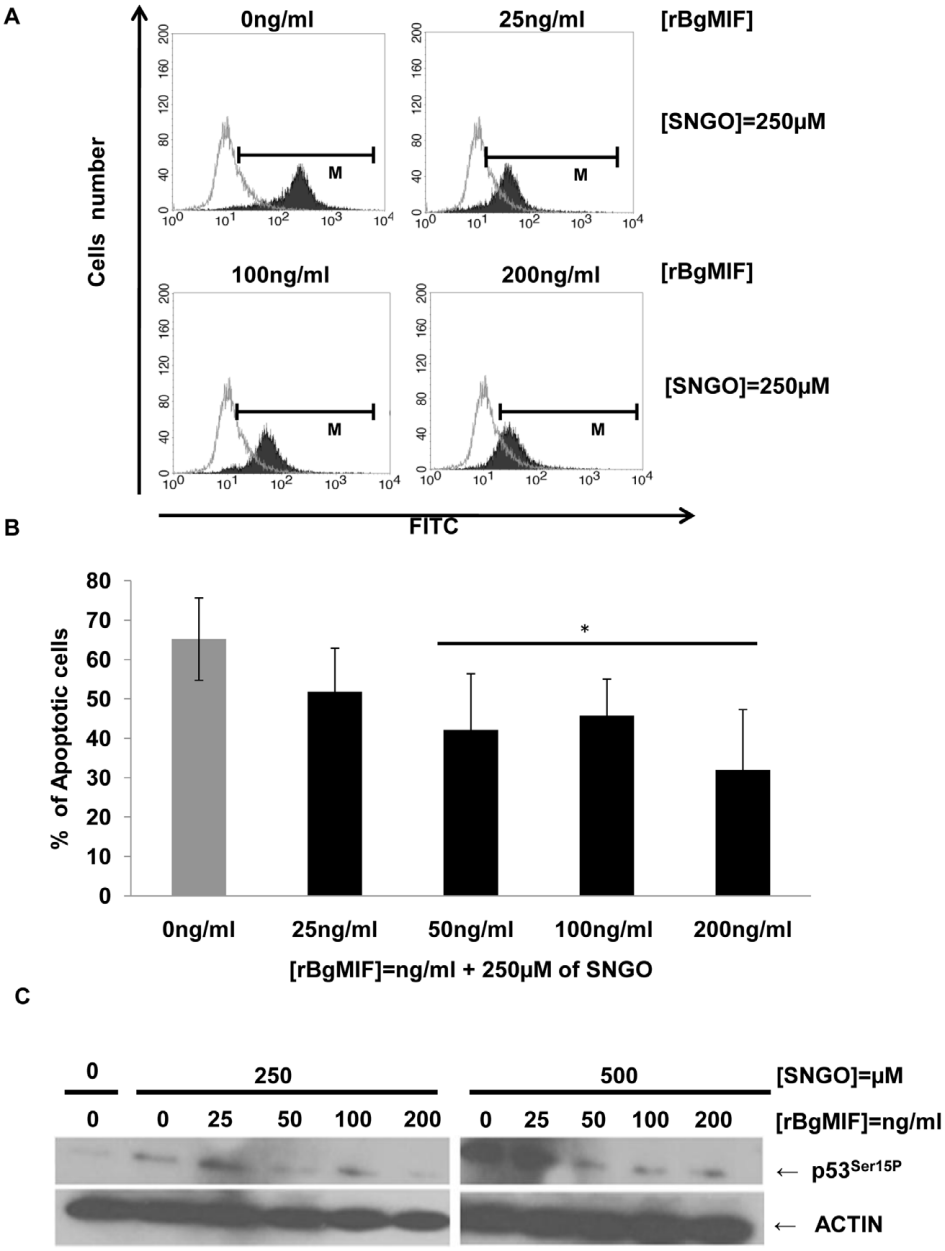
rBgMIF suppresses NO induced apoptosis in Bge cells. (A) Cells treated or not with the NO-donor SNGO were fixed and submitted to a TUNEL assay. The positively stained cells were quantified by FACS. (B) The FACS data are displayed as the percentage of cells undergoing apoptosis, i.e. cells within the limits of fluorescence shown in (A). Results are the mean ± SD of 3 separate experiments (*p<0.05). (C) Immunoblotting of cell lysates using an anti-p53^ser15P^ antibody (p53P^15^) and an anti-actin antibody to control for protein content in each sample.

MIF was found to inhibit NO-induced intracellular accumulation of p53 and, therefore, p53-mediated apoptosis in macrophages [31]. To investigate whether BgMIF inhibits apoptosis induced by NO accumulation, Bge cells were treated with the NO donor SNGO and different concentrations of rBgMIF. The proportion of apoptotic cells, labeled by the TUNEL method, was quantified using FACS analysis. As in mammalian macrophages, SNGO induced a significant level of apoptosis in Bge cells that was decreased in a dose-dependent manner by the addition of BgMIF (Figure 5A) and except for the lowest concentration of rBgMIF (25 ng/ml), the decrease in the percentage of apoptotic cells was statistically significant (Figure 5B). In mammalian macrophages, it has been shown that NO treatment is associated with a coordinate increase in the phosphorylation of p53 on Ser15, and that immunoblotting for phosphorylated p53 is a sensitive way of detecting the influence of MIF on intracellular p53 [31]. Examination of *B. glabrata* ESTs and genome sequences have allowed us to characterize a p53 ortholog (GenBank accession number: GU929337). We therefore examined whether inhibition of apoptosis in Bge cells treated with rBgMIF could be related to a decrease in NO-induced p53 accumulation in these cells. Western blot analysis of cell lysates using a phospho-specific (Ser15) anti p53 antibody showed that rBgMIF inhibited p53 phosphorylation in Bge cells and suggested that this mechanism participated in the suppression by rBgMIF of NO-induced apoptosis (Figure 5C).

### BgMIF promotes *S. mansoni* sporocyst encapsulation *in vitro*

**Figure 6:**
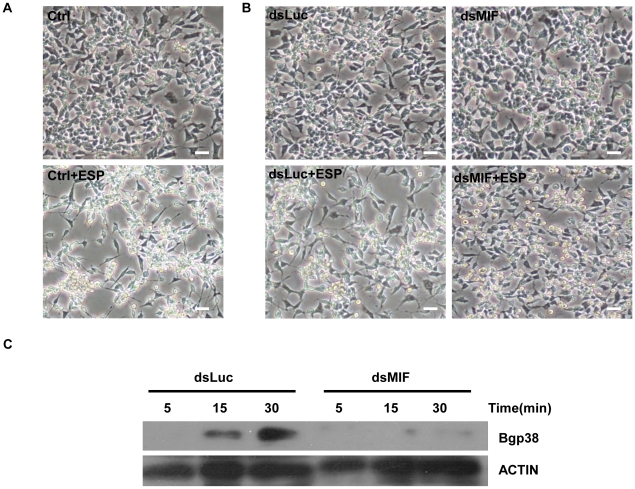
BgMIF promotes an aggregative phenotype and Bgp38 activation induced by ESP. (A) Effect of *S. mansoni* ESP (120 µg/mL) on the aggregative behavior of Bge cells. Cells incubated in the absence of ESP have a uniform distribution but cells incubated with ESP are clumped and their shape had changed. (B) Effect of *S. mansoni* ESP (120 µg/mL) on the aggregative behavior of Bge cells exposed to Luc (dsLuc) or BgMIF (dsMIF) dsRNA for 3 days and subsequently incubated or not with 120 µg/mL of sporocyst ESPs. Cells treated with dsLuc show normal aggregative behavior in the presence of ESP whereas those treated with dsMIF no longer aggregate in the presence of ESP. Note that the same number of Bge cells (2×10^5^) was used in each case. Scale bar represents 100 µm. (C) Effect of *S. mansoni* ESP (120 µg/mL) on the activation (phosphorylation) of Bgp38 of dsRNA treated Bge cells.

**Figure 7:**
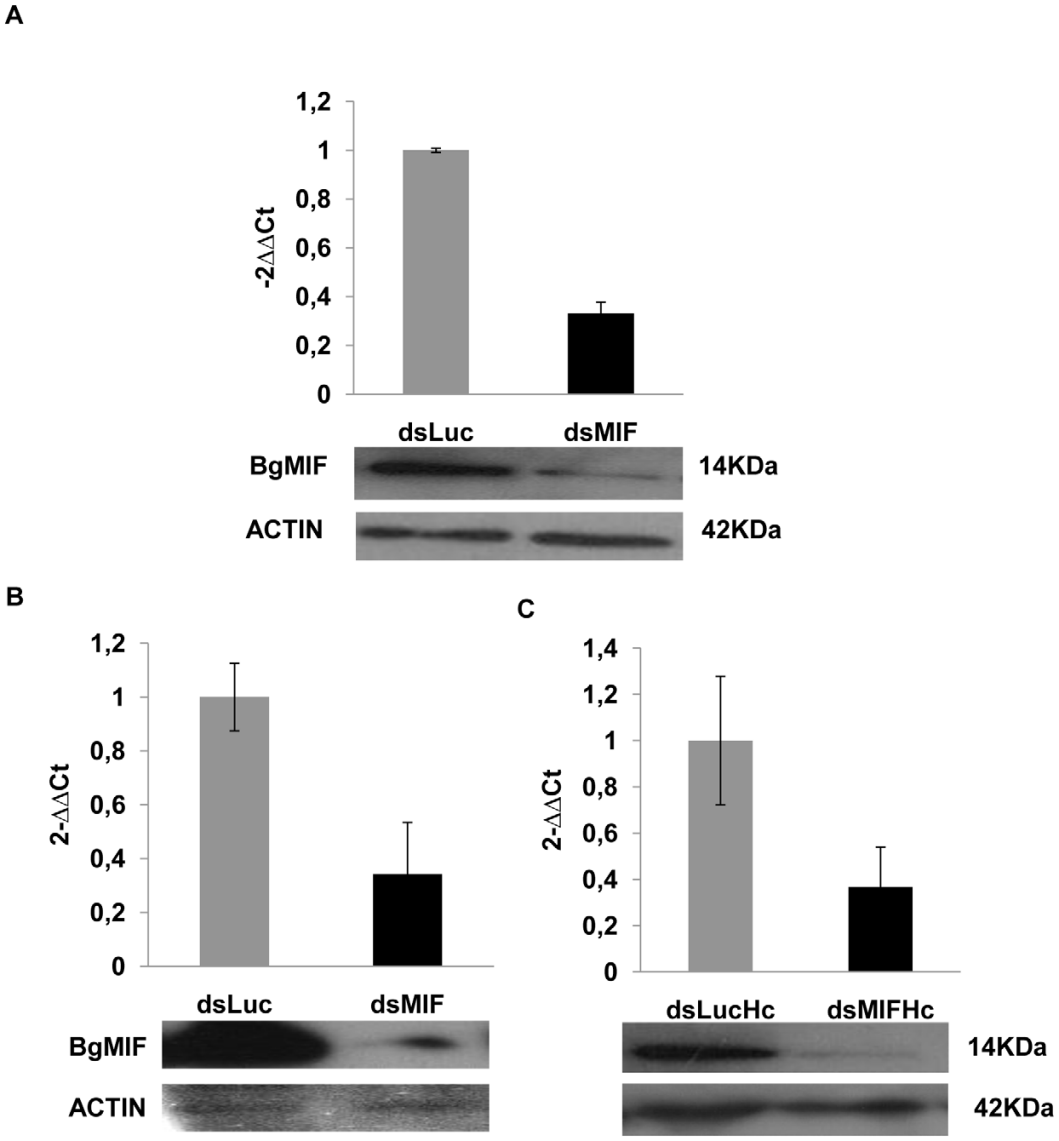
RNAi efficacy in Bge cells, whole snails and hemocytes. (A) Relative expression ratios of BgMIF transcripts and BgMIF protein in Bge cells exposed for 3 days to Luc (dsLuc) or BgMIF (dsMIF) dsRNA. Transcript levels were determined using real-time quantitative PCR (Methods) and protein levels of BgMIF and actin were determined by western blotting (B–C) Relative expression ratios of BgMIF transcripts and BgMIF protein in whole snails and in their hemocytes (Hc) exposed for 3 days to Luc (dsLuc) or BgMIF (dsMIF) dsRNA. Transcript levels were determined using real-time quantitative PCR (Methods) and protein levels of BgMIF and actin were determined by western blotting.

We have demonstrated that Bge cells secrete BgMIF when they are incubated with *S. mansoni* ESP (Figure 3H). In these experiments, we additionally observed that Bge cells aggregated and changed their form upon ESP induction (Figure 6A Ctrl+ESP) like mammalian macrophages induced by LPS [32], nevertheless this phenotype had not previously been described for *B. glabrata* cells and was not due to contaminating endotoxin in the ESP preparation (see Methods). In order to determine whether this aggregative behavior was regulated by BgMIF, we used RNAi to knock-down (KD) its expression in Bge cells, using dsRNA against BgMIF (dsMIF) or dsRNA against luciferase (dsLuc) as an unrelated control. The efficiency of BgMIF KD was confirmed by the marked decrease (70%) of BgMIF transcripts observed after a 3 day incubation with dsMIF, as compared to incubation with dsLuc (Figure 7A). When *S. mansoni* ESP (120 µg/mL) was added to cells treated with dsRNA, aggregation was observed in dsLuc (Figure 6B dsLuc+ESP) but not in dsMIF treated cells, which remained well-individualized, with numerous round and unspread refringent cells (Figure 6B dsMIF+ESP), suggesting that BgMIF is involved in the regulation of Bge cell activation induced by parasites. *S. mansoni* ESP have been shown to stimulate the p38 MAPK signaling pathway in Bge cells [33] manifested by the phosphorylation of Bgp38. We therefore tested the increase in phosphorylation of Bgp38 in response to the incubation with ESP. We detected a rapid activation of Bgp38 after 15 min in dsLuc treated cells, while in dsMIF treated cells p38 was not activated (Figure 6C).

**Figure 8:**
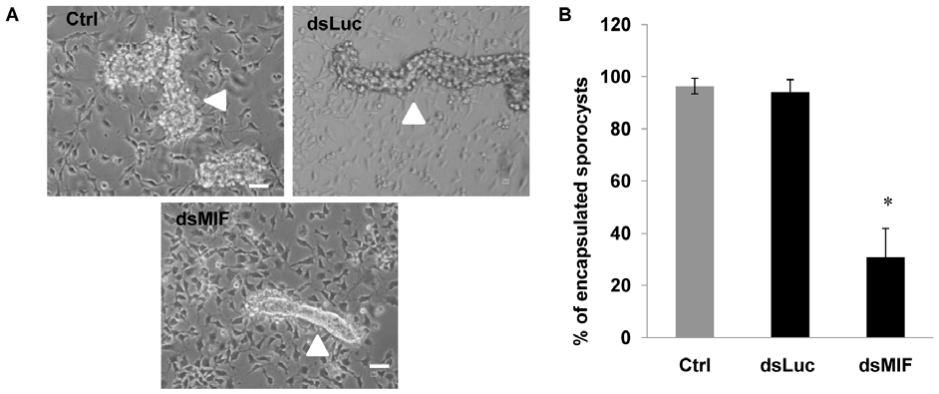
BgMIF is essential to promote *in vitro* encapsulation of *S. mansoni* sporocysts. (A) Encapsulation response of Bge cells in the presence of *in vitro* transformed *S. mansoni* sporocysts. Cells were exposed to Luc (dsLuc) or BgMIF (dsMIF) dsRNA. Sporocysts are indicated with an arrow. Note that, in the dsMIF culture, the sporocyst tegument remained easily visible and free of cells whereas in the control cultures, sporocysts became uniformly covered with Bge cells. (B) Quantification of sporocysts encapsulated by dsRNA-treated or untreated Bge cells. Sporocysts were deemed not to be encapsulated if Bge cells were found adhering to less than 10% of their surface. Scale bar represents 10 µm. The results are expressed as the percentage of sporocysts that were encapsulated and are the mean ± SD of three independent experiments.

We next determined the effect of BgMIF-induced cell activation using the *in vitro* model of *S. mansoni* sporocyst encapsulation by Bge cells [27], [34]. To Bge cells treated with dsMIF or dsLuc for 72 h, we added 48 h *in vitro*-transformed sporocysts and followed interaction of Bge cells with sporocysts for a further 72 h. Control (as well as dsLuc treated) Bge cells (Figure 8A) readily migrated towards and encapsulated the sporocysts as previously observed [34] but dsMIF-treated cells showed a markedly reduced ability to encapsulate the sporocysts (Figure 8A). The proportion of encapsulated sporocysts was indeed significantly reduced (*p*<0.05) in dsMIF treated Bge cells compared to dsLuc treated cells or untreated control cells (Figure 8B).

### BgMIF is implicated in hemocyte activation in response to parasitic infection *in vivo*

**Figure 9:**
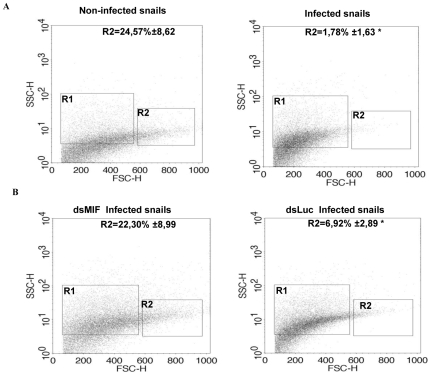
BgMIF is implicated in hemocyte activation in response to parasitic infection *in vivo*. (A) Profile of circulating hemocyte populations in non-infected or infected *B. glabrata* 24 h after infection with 15 *S. mansoni* miracidia. Two major hemocyte subpopulations (R1 =  small and medium and R2 =  large) can be identified by flow cytometric dot plot distribution based on laser forward scatter (FSC) versus laser side scatter (SSC). Mean percentages of the R2 population +/− S.D. in three separate experiments is shown. (B) Effect of *S. mansoni* infection on the circulating hemocyte populations of dsLuc or dsMIF treated snails. Mean percentages of the R2 population +/− S.D. in three separate experiments is shown. *designates values that differ significantly from the R2 population of non infected snails (*p*<0.05).

Since BgMIF promotes cellular responses to immune stimulation *in vitro,* we examined its role in the activation of hemocytes in *B. glabrata* snails confronting a parasitic infection. We first analyzed the circulating hemocyte population in non-infected versus infected snails, using flow cytometry based mainly on size (forward scatter-FSC) and granularity (side scatter-SSC) dot plot distribution. In non-infected snails the content of circulating hemocytes was shown to be very heterogeneous and we could discriminate two subpopulations, R1 (small and medium hemocytes) and R2 (large hemocytes) (Figure 9A). 24 h following infection, the population of circulating hemocytes showed a marked reduction in the R2 subpopulation of large cells or granulocytes (Figure 9A), which together with hyalinocytes, make up the heterogenous cell population present in healthy snails [25]. This decrease in circulating granulocytes is linked to their migration toward the tissues invaded by miracidia [35], [36].

In order to determine whether these cellular changes were regulated by BgMIF, we performed RNAi KD in whole snails by microinjecting 15 µg dsMIF or dsLuc into the cardiac sinus. BgMIF expression was monitored three days after dsRNA injection. We observed a decrease in BgMIF transcripts and protein in both whole snails and circulating hemocytes (Figure 7B–7C) treated with dsMIF, as compared to dsLuc treated animals. Next we infected control and dsMIF or dsLuc-treated snails by *S. mansoni* miracidia and analyzed the circulating hemocyte content 24 h after infection. Compared to dsLuc controls, dsMIF-treated infected snails exhibited a hemocyte profile similar to that found in non-infected snails (Figure 9B). These results corroborated the data obtained *in vitro* with Bge cells and further supported a role for BgMIF in the hemocyte response during a parasite infection of *B. glabrata*.

**Figure 10:**
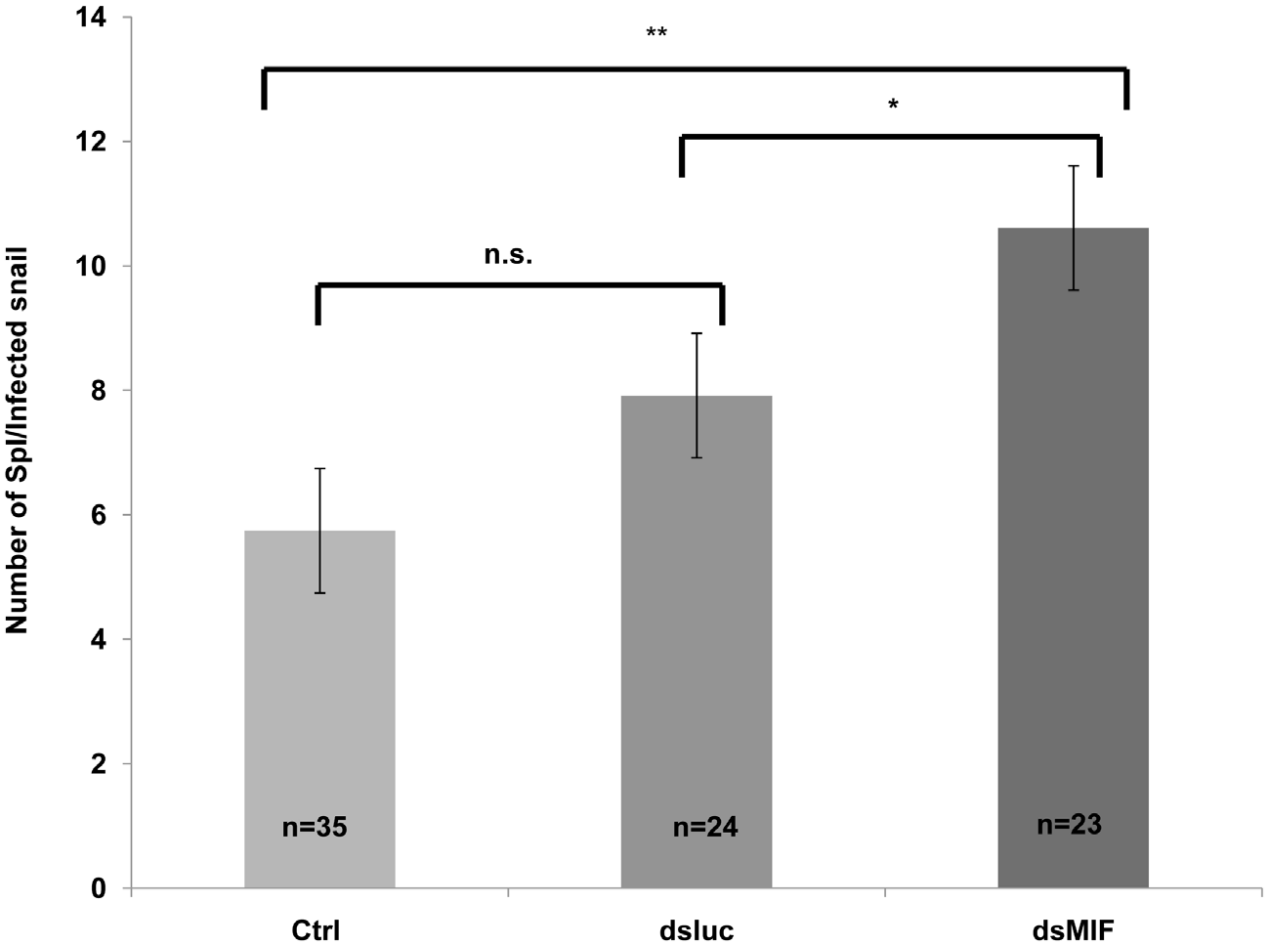
BgMIF controls the infectivity of *S.mansoni*. Effect of knockdown of BgMIF on *S. mansoni* infectivity. Snails were injected with both dsLuc or dsMIF dsRNA and 72 h later, were infected with 20 miracidia. 15 days after infection snails were fixed in Raillet–Henry's solution and dissected and visually inspected to detect mother sporocysts (Sp1). Results from two independent experiments are shown. *(p<0.05) or ** (p<0.01) designates values that differ significantly in the dsMIF injected snails compared to dsLuc injected or untreated (Ctrl) snails.

Finally, we tested the effect of BgMIF silencing of the snails on the level of infection observed with *S. mansoni* miracidia. We observed that dsMIF treated snails have significantly more parasites than dsLuc treated and control, untreated snails (Figure 10). These results further show that BgMIF is essential for the control of the immune response of snail against parasite infection.

## Discussion

Invertebrate immune systems have now become a major research focus for investigating broader questions such as the diversity of immune responses including those against parasitic and viral infections [37], [38], processes involved in the immunity of lophotrochozoan invertebrates, or the question of cytokine-dependent regulatory processes [9], [39]. Here we investigated the role of MIF, a putative ortholog of the vertebrate cytokine in the immune response of a lophotrochozoan invertebrate, the gastropod *Biomphalaria glabrata* towards its natural parasite *Schistosoma mansoni*.

The BgMIF protein sequence shares sequence and structural homologies with other members of the MIF family, including residues that are invariant across the whole MIF family (Asp9, Pro 56 and Leu88), or involved in the tautomerase enzymatic activity (Pro2, Lys32, Ile64). In addition, analysis of the secondary structure shows that the BgMIF protein is composed of four α-helices and four β-sheets as for other MIF family members. Results obtained with the recombinant BgMIF proteins indicated that BgMIF has a conserved dopachrome tautomerase enzyme activity dependent on the Pro2 residue and the results obtained with ISO-1, which interacts with the catalytic active site residues of mammalian MIF [22], show that the catalytic active site of BgMIF is conserved (even though residues Tyr 95 and Asp 97 in human MIF are respectively Val (as in the tick *I. scapularis*, Figure 1A) and Lys (as in the abalone MIFs) in BgMIF). The requirement for the enzymatic activity of MIF proteins for their biological activity has not been established and the use of a tautomerase-null *MIF* gene knock-in mouse model indicated that it was not involved in the growth-regulatory activity of the cytokine [40]. However, it does appear that the catalytic site could be important for pro-inflammatory activity, but the reason for this is still unknown. One possibility is that the catalytic site of MIF affects its binding to the MIF receptor, CD74, and its activation [41]. It has also been suggested that the tautomerase activity may be a vestige of a role in the invertebrate melanotic encapsulation response against microbial invasion, reflecting an ancestral role of the protein in the innate immune response [42]. In the context of BgMIF the significance of this tautomerase activity remains unknown as is the hypothetical interaction of the catalytic site with an as yet uncharacterized BgMIF receptor. On the other hand, the immunological relevance of this activity does not seem to be related to melanotic encapsulation, since melanization has not been described for *B. glabrata*.

In contrast to most cytokines, MIF is constitutively expressed and stored in intracellular pools and does therefore not require *de novo* protein synthesis before release into the extracellular milieu. These features provide MIF with the capacity to be released immediately and to act as an effector molecule regulating innate immunity [10]. Macrophages, which represent the first cellular barrier of defense towards pathogen invasion, are an important source of MIF protein, but MIF is also expressed in the tissues that are in contact with the host's natural environment. In this study we showed that BgMIF protein was constitutively present in all the snail tissues tested. In addition we found BgMIF protein in the cytoplasm of hemocytes. It was more abundant in the granulocytic forms that are involved in defence responses including phagocytosis or encapsulation of pathogens, than in the cytoplasm of hyalinocytes. In addition we have demonstrated the presence of BgMIF protein in the plasma of snails and its decrease after infection by *S. mansoni*. This decrease correlates with the migration of granulocytes towards infected tissues. We further found BgMIF protein in the cytoplasm of Bge cells that share hemocyte characteristics. Bge cells also secreted BgMIF protein in the presence of *Schistosoma* released products, thus validating the use of these cells in the bio-assays we developed to assess the biological activities of recombinant BgMIF.

When they enter *B. glabrata* snails, *S. mansoni* miracidia are readily located by the hemocytes and trigger marked cellular and humoral responses involving both migration of hemocytes toward the site of infection and an increase in hemocyte production [43]. In addition, reactive nitrogen and oxygen species (RNS and ROS) produced by the hemocytes can damage miracidia/newly-transformed sporocysts [38], but at the same time they can also induce hemocyte apoptosis. Important biological activities of MIF are likely to play a key role in this immune response, including the activation of MAPK such as ERK1/2 and the inhibition of p53-mediated apoptosis [10]. Although the MAPK pathways have only been partially characterized in mollusks, previous studies on *B. glabrata* have documented the involvement of ERK1/2 and other MAPK family members in signaling events leading to cellular immune responses in this snail [33], [44], [45], [46]. Using Bge cells as an *in vitro* model, we demonstrate in this work the induction of cell proliferation by rBgMIF in a dose-dependent manner. This proliferation was correlated with an increase in phosphorylated ERK in Bge cells as early as 2 h that was sustained for at least 24 h. This activity seems directly linked to the proliferation of the defense cells in response to immune stimulation.

The inhibition of NO-induced intracellular p53, which in turn inhibits apoptosis, is a well-documented effect of mammalian MIF that leads to a sustained proinflammatory function in macrophages exposed to LPS [31]. We showed that incubation with rBgMIF significantly reduced the apoptotic response of Bge cells, induced by SNGO as an NO-donor. The inhibition of apoptosis was accompanied by a reduction in the amount of phosphorylated Bgp53, the p53 ortholog recently identified in *B. glabrata* snails. These data further support the conservation of essential functions of BgMIF that enable it to regulate the immune response in this invertebrate.

BgMIF thus presents conserved activities of the MIF family and in order to address the question of its involvement in the innate immune response we first determined its role in the interaction of Bge cells with the sporocyst larvae of *S. mansoni*. We have optimized the KD of BgMIF transcripts and showed for the first time that the reduction of protein level was related to a resulting phenotype *in vitro*. When Bge cells were stimulated with *S. mansoni* ESP, they acquired an activated phenotype (aggregation) that was no longer observed in cells treated with dsMIF. In addition we showed that this aggregative phenotype was correlated with phosphorylation of Bgp38, an activation known to promote cell adhesion [33]. Using the *in vitro* co-cultivation system of Bge cells and *in vitro*-transformed sporocysts [27], we observed that the KD in BgMIF expression led to fewer Bge cells migrating towards and encapsulating the sporocysts, resulting in a lower percentage of encapsulated sporocysts than observed in control conditions. These results suggest that BgMIF is intimately involved in the response of the snail to parasites.

We next performed BgMIF KD *in vivo* using the microinjection of dsRNA previously described for *B. glabrata* snails [47] and analyzed its consequences on the hemocyte population of snails exposed to infection by *S. mansoni* miracidia. After 24 h following infection, the population of circulating hemocytes in non-interfered snails showed a marked reduction in the number of large cells (granulocytes), which, together with hyalinocytes, form the heterogeneous hemocyte population present in healthy snails [25]. This decrease in circulating granulocytes is due to their mobilization by the parasites and their migration towards the invaded tissues [35], [36]. When we examined the hemocyte populations of infected dsMIF-treated snails, we did not observe such a reduction in the number of granulocytes, suggesting that BgMIF is necessary for hemocyte activation during the *in vivo* response to the parasite. This change in the behavior of hemocytes was accompanied by a significant increase in the number of sporocysts establishing in dsMIF-treated snails, underlining the importance of MIF in regulating the innate immune response toward the parasite. The absence of hemocyte activation may be due to the lack of a signal generated by BgMIF released under normal conditions. It has been demonstrated that MIF deficient macrophages are hyporesponsive to stimulation by LPS or Gram-negative bacteria stimulation and that this is due to TLR4 downregulation [48]. In dsRNA-treated snails we also showed that the absence of hemocyte migration correlated in these cells with a down regulation of a Toll receptor ortholog, BgToll1, which we have recently identified (unpublished data). These data suggest that the BgToll1 expression may be regulated by BgMIF and that BgMIF facilitates the activation of hemocytes and their migration towards invaded tissues.

Taken together, the results presented here demonstrate the involvement of BgMIF in the innate immunity of *B. glabrata*. This is the first functional study of a molecule involved in the regulation of the anti-parasite response in *B. glabrata*, and the tools developed here pave the way towards a better understanding of the complex interactions between medically important helminths and their molluscan snail hosts.

## Materials and Methods

### Ethics statement

All animal experimentation was conducted following the Nord-Pas de Calais Region and the Pasteur Institute of Lille guidelines for housing and care of laboratory animals and performed in accordance with institutional regulations after review and approval by the Nord-Pas de Calais Region (Authorization No. A59-35009) and Pasteur Institute (Authorization No. AF/2009) Animal Care and Use Committees.

### Animals and cells

Adult (6–10 mm in diameter) *B. glabrata* snails (albino strain), were raised in pond water and fed *ad libitum*. A Puerto-Rican strain of *S. mansoni* was maintained by passage through *B. glabrata* snails and *Mesocricetus auratus*. Miracidia were isolated from infected hamster livers and maintained in complete Chernin's balanced salt solution [49] (CBSS supplemented with 1 mg/ml glucose and 1 mg/ml trehalose) for 48 h to achieve *in vitro* transformation into mother sporocysts as described in [50]. Mother sporocysts and/or excretory-secretory products (ESP)-containing CBSS were then collected and used. The *B. glabrata* embryonic (Bge) cell line (ATCC CRL 1494; Rockville, MD), was maintained at 26°C under normal atmospheric conditions in complete Bge medium [51], supplemented with 10% fetal bovine serum (FBS; Sigma), and antibiotics (100 U/ml penicillin G; 0.05 g/ml streptomycin sulphate, 25 µg/ml amphotericin B, Sigma).

### RNA, cDNA and protein extraction

Total RNA and protein from individual snails was extracted using the TRIZOL reagent (Invitrogen) according to the manufacturer's instructions. Total RNA from Bge cells was extracted using the Rneasy Mini kit (Qiagen) according to the manufacturer's instructions. For hemocytes the collected hemolymph [52] was divided in two tubes for extraction of protein and for extraction of RNA. For cDNA synthesis, RNA from whole snails (1 µg) and Bge cells (0.1 µg) were used for reverse transcription using SuperScript III reverse transcriptase (Invitrogen) and the oligo(dT)_20_ primer.

### Molecular cloning of BgMIF and vectors

A partial cDNA sequence (EST GenBank accession number: CK989824[21]) was used to design specific primers and perform 5′ and 3′ RACE amplification (SMART RACE cDNA Amplification kit, Clontech) according to the manufacturer's instructions. The complete BgMIF coding sequence was then amplified using primers containing XhoI and XbaI restriction sites respectively (see Supplementary data Table S1 for primer sequences). The PCR products were digested and cloned into the bacterial expression vector pET303 Ct-His (Invitrogen) (BgMIF construct). A BgMIF mutant construct (P2G pET303 Ct-His) was generated by site directed mutagenesis using primers (Supplementary data Table S1) encoding glycine instead of proline after the initiating methionine (BgMIFP2G construct). Sequence alignments and analysis were carried out using the DNAStar Lasergene programme package and the BioEdit v7.0.1 package (http://www.mbio.ncsu.edu/BioEdit/bioedit.html).

For phylogenetic analysis, multiple amino acid sequence alignments were performed using MUSCLE [53] or 3DCoffee [54]. The maximum likelihood tree was obtained with PhyML 3.0 [55] or MrBayes [56] at LIRMM (http://www.phylogeny.fr/) using the WAG model of substitution with four substitution rate categories and estimated gamma shape parameter and proportion of invariant sites. Branch support values were based on 500 bootstrap replicates with PhyML or 100000 replicates for MrBayes.

### Protein expression and production of recombinant proteins

Recombinant C-terminally His-tagged full-length rBgMIF and rBgMIFP2G fusion proteins were expressed using the pET303 Ct-His vector in *E. coli* BL21 (DE3) pLys strain. One liter of bacterial culture was grown to an OD_600 nm_ of 0.4 and induced by addition of isopropyl β-1-D-thiogalactopyranoside to a final concentration of 0.4 mM. After 3 h at 30°C, cells were harvested, lysed and purified with Ni-NTA agarose resin (Qiagen) according to the manufacturer's instructions. Briefly, cells were resuspended in lysis buffer (50 mM NaH_2_PO_4_, 300 mM NaCl, 10 mM imidazole) and disrupted by successive freeze-thaw cycles in liquid nitrogen. The soluble protein fraction was mixed with Ni-NTA agarose and incubated under agitation for 1 hour at 4°C. The resin was then washed (50 mM NaH_2_PO_4_, 300 mM NaCl, 20 mM imidazole) and finally tagged proteins were eluted with 50 mM NaH_2_PO_4_, 300 mM NaCl, 200 mM imidazole. The purified protein was dialyzed against endotoxin-free PBS overnight and the content of remaining endotoxin was measured with Limulus Amoebocyte Lysate (Cambrex). Recombinant proteins used in the bioassays contained less than 200 pg endotoxin/mg of protein.

### Enzymatic assay

Tautomerase activity was measured using a D-dopachrome tautomerase assay as described previously [14], [22]. Briefly, a fresh solution of D-dopachrome methyl ester was prepared by mixing 4 mM L-3,4-dihydroxyphenylalanine methyl ester with 8 mM sodium periodate for 5 min at room temperature that was then placed on ice 20 min before use. Activity was determined at room temperature by adding D-dopachrome methyl ester to a cuvette containing 50 mM rBgMIF, BgMIFP2G or a commercial mammalian MIF (mouse MIF), rMmMIF (R&D systems), in 25 mM potassium phosphate buffer pH 6.0 and, 0.5 mM EDTA. For the inhibition assays the MIF inhibitor, (*S,R*)-3-(4-hydroxyphenyl)-4,5-dihydro-5-isoxazole acetic acid methyl ester (ISO-1, Merck) was dissolved in Me_2_SO at various concentrations and added to the cuvette with rBgMIF prior to the addition of the dopachrome. The decrease in absorbance at 475 nm was monitored for 5 min using a UV/visible Spectrophotmeter Ultraspec 2100 (Amersham).

### Antibodies and Western-blots

Antibodies used in this study were as follows: anti-Actin (Abcam), anti-phospo-p53^Ser15^, anti-phospho-p44/42 MAPK (Erk1/2) (Thr202/Tyr204), anti- p44/42 MAPK (Erk1/2) and anti-Phospho-p38 MAPK (Thr180/Tyr182) (Cell Signaling). An anti BgMIF antiserum was produced in a rabbit using the Ac-HKDITEIASEFLQKSQK-amide, Ac-VRVPALFCQFDGHLHGH-amide peptides and the polyclonal sera were purified using a peptide linked resin column (Proteogenix). For western blot analysis cells or snails were lysed in Tris-buffered saline (50 mM Tris-Base, 150 mM NaCl, pH 7.5) containing 1% NP-40, 0.5% deoxycholic acid, 0.1% SDS, 2 mM EDTA, and 1 mM PMSF), cellular debris was pelleted and the supernatants were adjusted for protein concentration and diluted with reducing SDS-PAGE sample buffer. For BgMIF analyses the total protein extracts were separated by SDS-PAGE in pre-cast 16% tricine gels (Invitrogen) and transferred to a PVDF membrane with 0.2 µm pore size (Millipore). For actin, ERK, p38 and p53 analyses the total protein extracts were separated by SDS-PAGE Tris-glycine gels and transferred to a PVDF membrane with 0.45 µm pore size (Millipore). Western blot analyses were then performed using the SNAP id system (Millipore) according to the manufacturer's instructions.

### Expression studies

For real-time PCR analyses total RNA (1 µg of RNA from a 5 snail pool and one tenth of the RNA obtained from 2×10^5^ Bge cells or collected haemocytes) was reverse transcribed using SuperScript III reverse transcriptase (Invitrogen). For Q-PCR analyses, cDNAs used as templates were amplified using the SYBR Green Master Mix (Invitrogen) and the ABI PRISM 7000 sequence detection system (Applied Biosystems). Primers (Sup. Table 1) specific for *B. glabrata* ribosomal protein S19 (Genbank accession number CK988928;[21]), and BgMIF, were designed by the Primer Express Program (Applied Biosystems) and used for amplification in triplicate assays.

The organ distribution of BgMIF protein was determined in adult snails. Organs (albumen gland, hepatopancreas, foot, heart and digestive tract) were excised, sectioned and homogenized at 4°C with Tris-buffered saline. The cellular debris was pelleted and the supernatants were adjusted for protein concentration, diluted with reducing SDS-PAGE sample buffer and 15 µg of total protein was analyzed by SDS-PAGE and western blotting as described above.

For the measurement of BgMIF protein contained in the plasma (cell free hemolymph) after infection we used an indirect ELISA protocol. Briefly, the cell hemolymph for five snails was pooled and centrifuged to pellet the cells. Wells of a PVC microtiter plate were coated with 100 µl of hemolymph diluted by half in PBS and plates were incubated for 2 h at room temperature. Then, after the coating solution was removed, plates were washed three times by filling the wells with 200 µL of PBS. The solutions or washes were removed by flicking the plate over a sink. The remaining protein binding sites in the coated wells were blocked by adding 200 µL of blocking buffer (5% bovine serum albumin (BSA) in PBS) per well. Plates were covered and incubated for 2 h at room temperature. Plates were then washed twice with PBS, and 100 µL of anti-MIF antibody (from rabbit, diluted 1/500, Proteogenix) diluted in blocking buffer was added to each well. Plates were covered and incubated overnight at 4°C. Subsequently, plates were washed four times with PBS, and 100 µL of conjugated secondary antibody (antirabbit, diluted 1/5000, Jackson Immuno Research) diluted in blocking buffer was added to each well. Then, plates were covered and incubated 2 h at room temperature. After four washes, the horseradish peroxidase (HRP) activity was measured using the colorimetric substrat TMB (3,3–5,5 –Tetramethylbenzidine) blue substrate (Roche Applied Science). A standard with rBgMIF ranging between 0–230 ng/ml was used. The measurements were made twice in triplicate with two different infection experiments using a microplate reader MRXII (Dynex Technologies) at 450 nm; the haemolymph was pooled before infection and 6, 24 and 48 h post infection.

### Immunolocalisation of MIF in cells

For immunolocalisation assays, circulating haemocytes or Bge cells were extracted or cultured as described above and allowed to adhere to glass slides, washed with PBS, fixed in 4% paraformaldehyde for 10 min and permeabilised by a 4 min treatment with Triton X-100 at 0.1%. Slides were saturated for 90 min with PBS containing 1% bovine serum albumin (BSA) and normal goat serum (1/50) at room temperature (RT). This blocking step was followed by an overnight incubation with rabbit anti-BgMIF polyclonal serum (diluted at 1/100 in PBS-BSA 1%). After three washes the slides were incubated with goat anti-rabbit Alexa Fluor 488 IgG (1/500 in PBS-BSA 1%, Molecular Probes) for 2 h at room temperature (RT). Slides were then stained with Hoechst 33342 and rhodamine-labeled phalloidin (1/1000 in PBS, Sigma) for 10 min at RT, washed and mounted with Fluoromont G (Interchim). For control slides, anti-BgMIF polyclonal serum was incubated with the peptides used as immunogens for 1 h at RT and the slides were then treated as described above. Samples were analysed by confocal microscopy using a LSM 710 inverted microscope (Zeiss).

### Confocal microscopy

All the confocal imaging was performed with a LSM710 microscope (Zeiss) and a Plan Apochromat objective (63×1.4 NA oil immersion). The associated software (Zen 2008) enabled the adjustment of acquisition parameters. The rhodamine (red) signal was excited at 561 nm and emission was collected from 570 to 700 nm. The Alexa488 (green) signal, in contrast, was excited at 488 nm and emission was collected from 490 to 530 nm. The nuclear Hoechst dye signal was excited at 405 nm and emission was collected from 410 to 470 nm. Fluorescent signals were collected sequentially, with a 4 lines average, and resulting images are 2048×2048 (or 1024×1024) pixels in size. By setting the photomultiplier tubes and the pinhole size (1 AU) correctly, there was no signal bleed-through. The images were treated with ImageJ (NIH) and Photoshop CS3 (Adobe).

### Proliferation and ERK MAPK activation studies

The protocol used was adapted from [29]. Briefly Bge cells (2×10^4^ cells/well) were cultured until semi-confluent in 96-well plates in complete Bge medium. The cells then were synchronized by culture in 0.5% FCS-containing Bge medium overnight. The medium was then replaced by fresh medium (control condition) or medium containing different concentrations of rBgMIF. After incubation with rBgMIF the cells were pulsed with 10 µM of BrdU (Sigma) for 2 h and the proliferation was measured by ELISA method as described in [57]. In order to test the effect of inhibition of the ERK pathway, cells were treated with the MEK inhibitor U0126 (Cell Signalling) at 10 µM or Me_2_SO (solvent) for 30 min prior to the addition of rBgMIF and were then treated as above.

For analysis of sustained activation of ERK, cells (2×10^5^cells/well) were cultured and synchronized as described above. Cells were exposed to various concentrations of rBgMIF, for 2 h, 8 h and 24 h, and then lysed as described above. Cell lysates were used for western blot analysis of phosphorylated and total ERK content.

### Apoptosis assay

The apoptosis assay used was adapted from [31]. Bge cells (semi-confluent) were cultured in 6-well plates in complete Bge medium. Cells were pretreated for 12 h with rBgMIF at different concentrations. The NO donor, *S*-nitrosoglutathione (SNGO, Sigma) was then added at 250 µM for 8 h. Apoptosis was measured by the Terminal deoxynucleotidyl transferase mediated dUTP Nick End Labeling (TUNEL) assay (Roche Applied Biosystems), following the manufacturer's instructions. Briefly, the cells were fixed in paraformaldehyde 4% for 1 h, washed and permeabilized with sodium citrate 0,1%/Triton -X 100 0.1% for 2 min on ice. Cells were incubated with “labeling solution” for 1 h at 37° C, washed with PBS and the number of positive cells was visualized on a FACSCalibur flow cytometer (Becton Dickinson) and the data were treated with CellQuestPro software (Becton Dickinson). The data are displayed using a logarithmic scale and the results are represented as the percentage of cells undergoing apoptosis. Activation of p53 was investigated by performing a western blot on total protein extracts from Bge cells using the anti-p53^ser15P^ antibody and the anti-actin antibody.

### Production of dsRNA

PCR products were amplified from the pCR2.1 TOPO vector containing the complete BgMIF sequence, purified (Wizard SV Gel and PCR Clean up system, Promega) and used as a template for T7 transcription and synthesis of BgMIF dsRNA (MEGAScript T7 kit, Ambion). The firefly (*Photinus pyralis*) luciferase gene dsRNA (pGL3 vector, Promega) was used as a control (see Sup. Table 1).

### Stimulation of Bge cells with *S.mansoni* ESP

Bge cells (2×10^5^cells/well) were cultured in 12-well plates in complete Bge medium. For the analyses of BgMIF excretion the medium was changed for complete CBSS and the cells were stimulated with different quantities (30, 60, 120, 240 and 480 µg/ml of protein content) of *S. mansoni* ESP (prepared as described in [33]) for 12 h. Cell supernatants were collected, centrifuged for 10 min at 800 g to eliminate non adherent cells, then concentrated 10-fold by membrane filtration with a 10 kDa cut-off (Centricon, Amicon). For the study of Bgp38 activation, the protocol was adapted from [33]. Briefly, cells were exposed to 120 µg/ml of ESP for 5, 15 and 30 min, and the protein extracts were analysed for the phosphorylated p38 and actin content by western blotting. The content of endotoxin was measured with *Limulus* Amoebocyte Lysate (Cambrex). ESP used in the bioassays contained less than 17 pg endotoxin/mg of protein.

### RNA interference assays in Bge cells

Each dsRNA (2 µg) was transfected into confluent cultures of Bge cells using the FUGENE HD transfection reagent (Roche Applied Biosystems), following the manufacturer's instructions. For the experiments with EPS, the Bge medium was changed to complete CBSS 2 days after the addition of dsRNA. On the third day 120 µg/mL of ESP products were added to the medium and the presence or absence of an aggregation phenotype was determined. For encapsulation experiments, the medium was replaced by fresh medium and *S. mansoni* mother sporocysts cultured in complete CBSS for 48 hours, were added (500 sporocysts/well) 3 days after the addition of dsRNA. The co-culture was maintained 4 days to allow the observation of an *in vitro* encapsulation phenotype as described previously [50]. Aggregation and encapsulation phenotypes were observed using an Eclipse TS100 optical microscope (Nikon) and the images were acquired with a DS-Fi1digital camera (Nikon) and treated with Photoshop CS3 (Adobe). For encapsulation experiments, 250–300 sporocysts were counted per assay and the results were represented as the percentage of sporocysts completely covered in adhering cells.

### RNA interference assays in whole snails and FACS analysis

Each dsRNA (15 µg in 10 µl of sterile CBSS) was injected into the cardiac sinus of *B. glabrata* snails, using a 50 µl Hamilton syringe with a 26s needle (Hamilton). Three days after injection, hemocytes were isolated from three snails per group and the snails were individually frozen in liquid nitrogen for extraction of RNA and soluble protein. Knock down efficiency was checked by real-time PCR and western blot analyses. Snails were infected three days after injection, with 20 *S. mansoni* miracidia. 24 h post infection the hemolymph of three snails was pooled and diluted by half in complete CBSS containing citrate/EDTA (50 mM sodium citrate, 10 mM EDTA, and 25 mM sucrose) [58] and the composition of the hemocyte population in each condition was assessed by FACS analyses using SSC and FSC parameters in a FACSCalibur flow cytometer (Becton Dickinson). The hemocyte population was analyzed in pools of three snails.

In order to determine the number of mother sporocysts (Sp1) present in the head–foot region of infected snails, mollusks were fixed 15 days post-exposure as described [59], [60]. Briefly, the snails were relaxed in water containing an excess of crystalline menthol for 6 h. The snail shell was removed and the body was fixed in modified Raillet–Henry's solution (930 ml distilled water, 6 g NaCl, 50 ml Formol 40%, 20 ml acetic acid). The head–foot zone was dissected and visually inspected. In each snail Sp1 were readily observable as translucent white bodies within an opaque yellow tissue background.

### Statistical analyses

All data were expressed as mean plus or minus SE. The statistical significance of differences was assessed using the Mann–Whitney *U* test for nonparametric data or Student's *t*-test using the program StatView (Abacus Concepts). *P* values of less than .05, 0.01 or .001 were used to indicate statistical significance.

### Accession numbers

Nucleotide sequence data reported in this paper are available in the GenBank database under the accession numbers ACR81564 (BgMIF), GU929337 (Bgp53).

## Supporting Information

Figure S1 MIF antagonist ISO-1 inhibits BgMIF D-dopachrome tautomerase activity. Analysis of the D-dopachrome tautomerase enzymatic activity measured as the loss in absorbance at 475 nm and plotted against the concentration of 2-carboxymethylester-2,3-dihydroindole-5,6-quinone for 50 nM wild type rBgMIF in presence of ISO-1. Results shown are the means +/− S.D. of three independent experiments.
(0.09 MB TIF)

Figure S2 BgMIF concentrations in snail plasma decrease after *S. mansoni* infection. After 0 h, 6 h, 24 h and 48 h infection by *S. mansoni* miracidia BgMIF levels in snail plasma were quantified by indirect ELISA tests. Results are the means +/− S.D. of the triplicate of two different infection experiments (*p<0,05; **p<0,001).
(0.09 MB TIF)

Figure S3 The MEK inhibitor U0126 prevents the stimulatory effect of BgMIF on Bge cell proliferation. The proliferation rate of Bge cells treated with 10 µM of U0126 or DMSO (solvent) was measured in the presence of various concentrations of rBgMIF. Proliferation was assessed using BrdU incorporation measured by an ELISA assay. Results are represented as fold increase in BrdU incorporation as compared to incorporation in control cells. The results shown are the mean ± SD of two assays carried out in quadruplicate and are representative of 3 separate experiments *p<0,05.
(0.14 MB TIF)

Table S1 Oligonucleotide primers used in the study.
(0.04 MB DOC)
