# Supplementary figures and images for: Involvement of the Cytokine MIF in the Snail Host Immune Response to the Parasite Schistosoma mansoni
Source: PLoS Pathog. 2010 Sep 23;6(9):e1001115. doi: 10.1371/journal.ppat.1001115 (PMC2944803; doi:10.1371/journal.ppat.1001115)

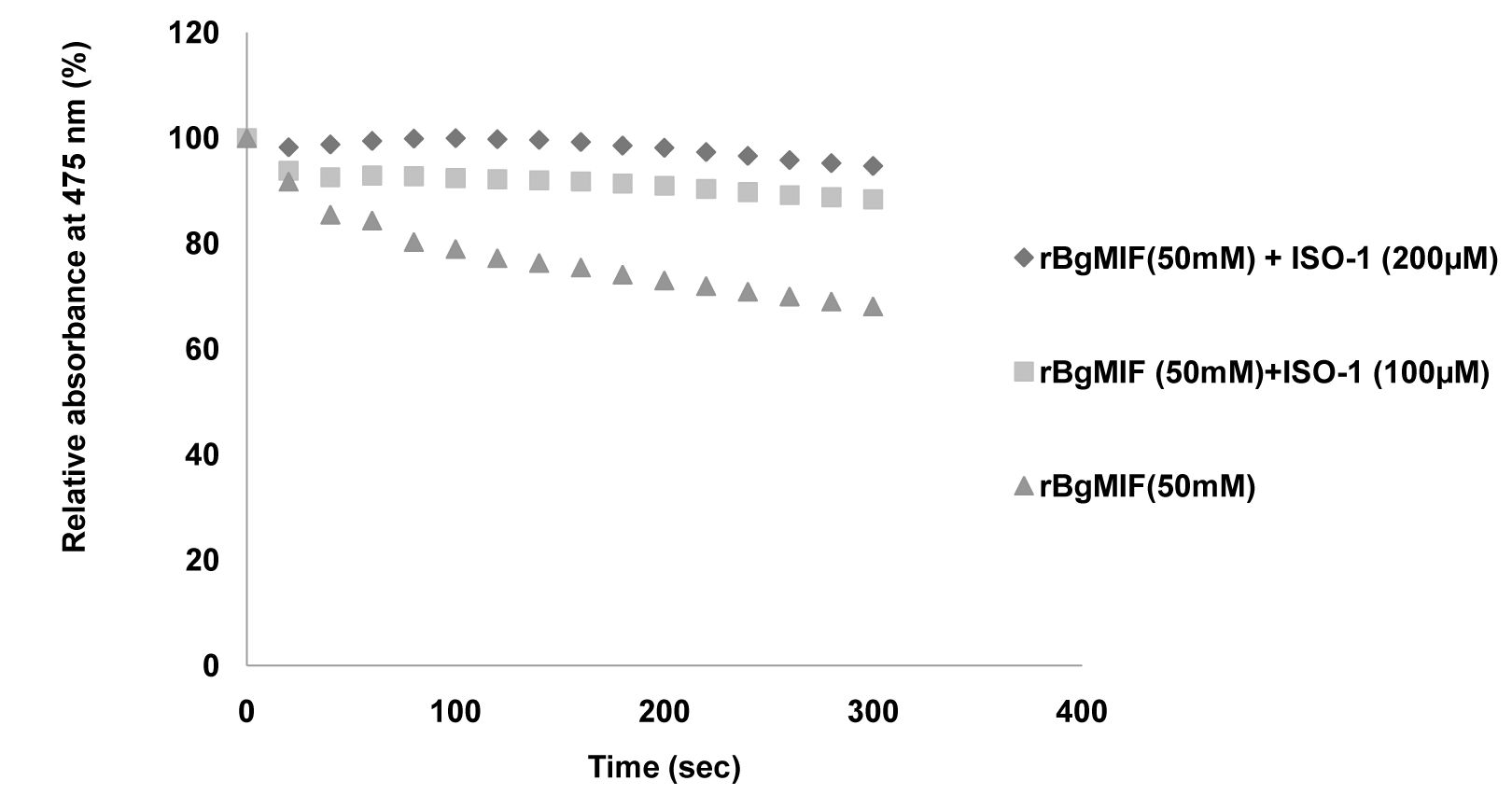

Supplement: Figure S1 — MIF antagonist ISO-1 inhibits BgMIF D-dopachrome tautomerase activity. Analysis of the D-dopachrome tautomerase enzymatic activity measured as the loss in absorbance at 475 nm and plotted against the concentration of 2-carboxymethylester-2,3-dihydroindole-5,6-quinone for 50 nM wild type rBgMIF in presence of ISO-1. Results shown are the means +/− S.D. of three independent experiments. (0.09 MB TIF) [file ppat.1001115.s001.tif]

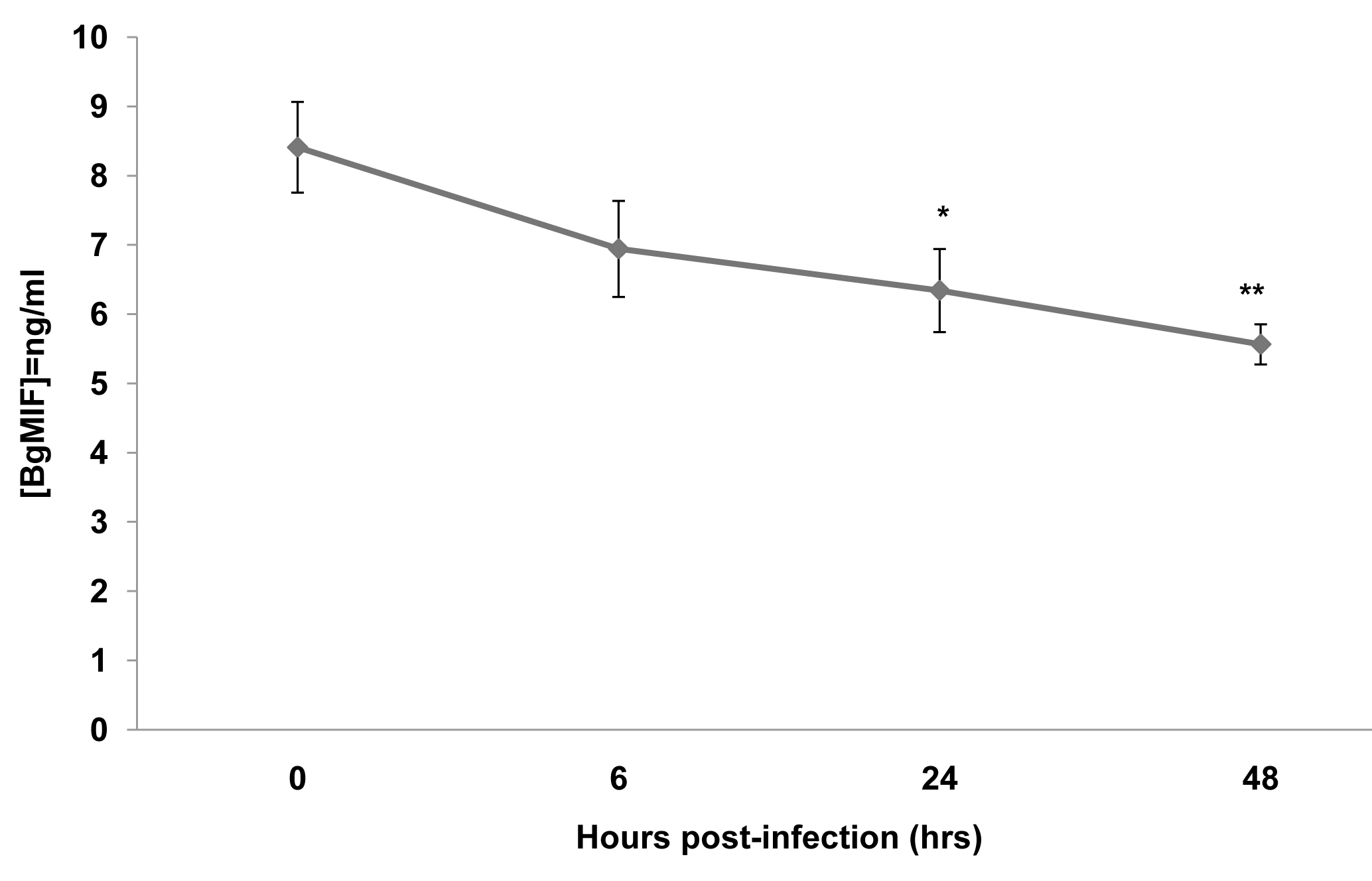

Supplement: Figure S2 — BgMIF concentrations in snail plasma decrease after S. mansoni infection. After 0 h, 6 h, 24 h and 48 h infection by S. mansoni miracidia BgMIF levels in snail plasma were quantified by indirect ELISA tests. Results are the means +/− S.D. of the triplicate of two different infection experiments (*p<0,05; **p<0,001). (0.09 MB TIF) [file ppat.1001115.s002.tif]

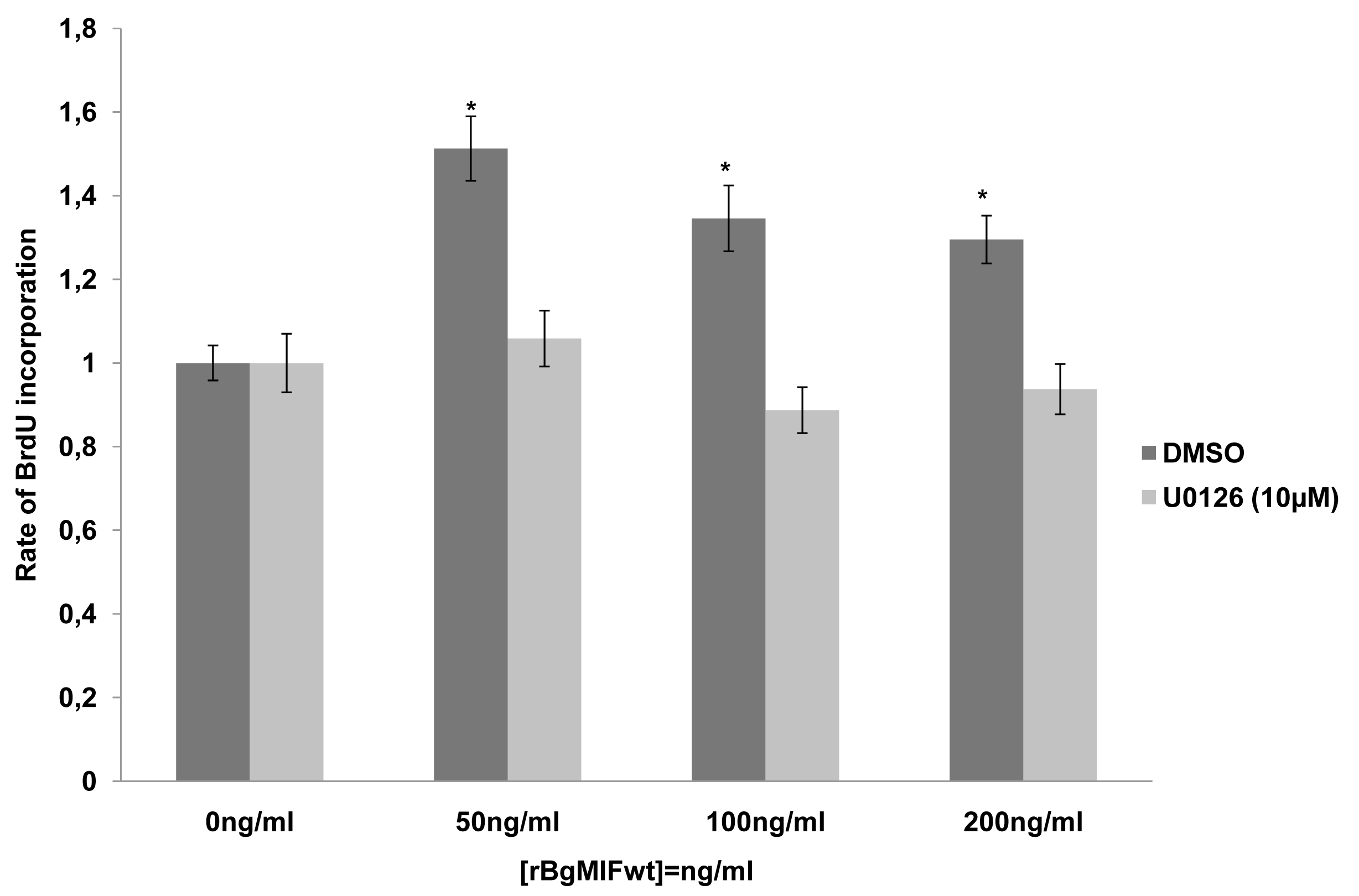

Supplement: Figure S3 — The MEK inhibitor U0126 prevents the stimulatory effect of BgMIF on Bge cell proliferation. The proliferation rate of Bge cells treated with 10 µM of U0126 or DMSO (solvent) was measured in the presence of various concentrations of rBgMIF. Proliferation was assessed using BrdU incorporation measured by an ELISA assay. Results are represented as fold increase in BrdU incorporation as compared to incorporation in control cells. The results shown are the mean ± SD of two assays carried out in quadruplicate and are representative of 3 separate experiments *p<0,05. (0.14 MB TIF) [file ppat.1001115.s003.tif]
