# Supplementary material for: Involvement of the Cytokine MIF in the Snail Host Immune Response to the Parasite Schistosoma mansoni
Source: PLoS Pathog. 2010 Sep 23;6(9):e1001115. doi: 10.1371/journal.ppat.1001115 (PMC2944803; doi:10.1371/journal.ppat.1001115)
Supplement: Table S1 — Oligonucleotide primers used in the study. (0.04 MB DOC) [file ppat.1001115.s004.doc]

**Supplementary Table 1**: **Oligonucleotide primers used in the study**

| **Gene** | **Primer Use** | **Sequence (5’-3’)** |  |
| --- | --- | --- | --- |
| BgMIF | Cloning in expression vector forward primer | CgCg**TCTAgA**CATgCCCATCATAACAATACAg |  |
|  | Cloning in expression vector reverse primer | gCgCTCA**CTCgAg**ATTCCAATgTCCATgAAg |  |
|  | Directed mutagenesis forward primer | ggAggtCTAgAATgggCATCATaacaatAc |  |
|  | Directed mutagenesis reverse primer | gTATTgTTATgATgCCCATTCTAgACCTCC |  |
|  | 3’RACE PCR primer | TGTCTCTGCTTGTCAAGCTGGTGCT |  |
|  | 3’RACE PCR nested primer | CCACAAAGACATCACAGAGATAGCCTCG |  |
|  | 5’RACE PCR primer | gCTCCCATAgTTAACTgTTTgTCTgTCTCCAgC |  |
|  | 5’RACE PCR nested primer | ggCTATCTCTgTgATgTCTTTgTggAAggAAg |  |
|  | Quantitative Rt-PCR forward | TGCCAGCCCTGTTCTGTCA |  |
|  | Quantitative Rt-PCR reverse | TCCCTTGAGGTCTTAATCAC |  |
|  | RNAi forward primer | CCATCATAACAATACAGAC |
|  | RNAi forward primer +T7 sequence | TAATACGACTCACTATAGGGAGACCATCATAACAATACAGAC |
|  | RNAi reverse primer | CCAATGTCATGAAGATGTC |  |
|  | RNAi reverse primer +T7 sequence | TAATACGACTCACTATAGGGAGACCAATGTCATGAAGATGTC |  |
|  |  |  |  |
| BgS19 | Quantitative Rt-PCR forward | tgctcgccacttgtacattc |  |
|  | Quantitative Rt-PCR reverse | gcccttgtggtgttagtcgt |  |
| Luciferase | RNAi forward primer | CTggAgACATAgCTTACTg |  |
|  | RNAi forward primer +T7 sequence | TAATACgACTCACTATAgggAgACTggAgACATAgCTTACTg |  |
|  | RNAi reverse primer | ggATCTCTCTgATTTTTCTTgCg |  |
|  | RNAi reverse primer +T7 sequence | TAATACgACTCACTATAgggAgAggATCTCTCTgATTTTTCTTgCg |  |

^a^ Restriction enzyme sites are in bold, start or stop codons are underlined.
